# Mood or energy/activity symptoms in bipolar mania: which are the most informative?

**DOI:** 10.47626/2237-6089-2022-0551

**Published:** 2024-08-14

**Authors:** Elie Cheniaux, Luis Anunciação, Jesus Landeira-Fernandez, Antonio Egidio Nardi

**Affiliations:** 1 Instituto de Psiquiatria Universidade Federal do Rio de Janeiro Rio de Janeiro RJ Brazil Instituto de Psiquiatria, Universidade Federal do Rio de Janeiro (UFRJ), Rio de Janeiro, RJ, Brazil.; 2 Universidade do Estado do Rio de Janeiro Rio de Janeiro RJ Brazil Universidade do Estado do Rio de Janeiro (UERJ), Rio de Janeiro, RJ, Brazil.; 3 Departamento de Psicologia Pontifícia Universidade Católica do Rio de Janeiro Rio de Janeiro RJ Brazil Departamento de Psicologia, Pontifícia Universidade Católica do Rio de Janeiro, Rio de Janeiro, RJ, Brazil.

**Keywords:** Bipolar disorder, motor activity, energy, mood changes, Young Mania Rating Scale

## Abstract

**Objective:**

In the DSM-III and the DSM-IV, bipolar disorder (BD) is classified as a mood disorder and diagnosis requires presence of a mood change, i.e., euphoria or irritability. In contrast, DSM-5 states that there must be some increase in energy or motor activity in addition to the mood change. Our aim was to identify which types of symptoms (i.e., mood or energy/activity-related symptoms) are the most informative in a manic episode.

**Methods:**

Symptoms of manic episodes in 106 outpatients with BD were assessed in a naturalistic study using the Young Mania Rating Scale (YMRS) between November 2002 and November 2015. The scale items were divided into three groups according to clinical criteria: mood, energy/activity, and other. For comparisons between groups, the Samejima graded response model from Item Response Theory (IRT) and the Test Information Function (TIF) were computed. Chi-square tests were used to verify the association between the groups of symptoms by comparing the area under the curve of the TIF results.

**Results:**

The information accounted for by energy/activity represents 77% of the proportion of the total TIF; about 23% is related to mood and other groups of symptoms. These proportions are statistically different (χ^2^[1] = 30.42, p < 0.001)

**Conclusion:**

On average, changes in energy/activity tend to be more informative than mood changes during the manic phases of BD.

## Introduction

Kraepelin^1^ defined manic and depressive syndromes based on three elements – mood, thought, and activity –, giving the same weight to all three types of symptoms. However, from the Diagnostic and Statistical Manual of Mental Disorders, 3rd edition (DSM-III),^2^ bipolar disorder (BD) was classified as a mood disorder, similarly to major depressive disorder, to the detriment of thought and activity.

In contrast to this view of BD as a mood disorder, Akiskal et al.^3^ proposed criteria for diagnosis of mania that emphasized increased motor activity. Several studies have also shown the importance of motor activity changes in mania. Factor analyses of manic symptoms found that the “hyperactivity” factor was the most heavily loaded factor.^3 - 6^ Additionally, some actigraphic studies have shown that patients with BD exhibit increased motor activity during manic episodes.^7 - 10^

Probably as a result of the findings of these studies, the DSM-5^11^ introduced an important change to criterion A for diagnosis of a manic or hypomanic episode. Up to the DSM-IV-TR,^12 , 13^ euphoria or irritability had been mandatory. Nevertheless, increased energy or motor activity must now be accounted for in addition to mood symptoms. This obviously implies a modification to the concept of BD.

It is still debatable whether changes in mood and in energy/motor activity have the same level of importance in BD, particularly in mania. In the present study, we compared the two kinds of symptoms during manic episodes in outpatients with BD. We used Test Information Function (TIF) analysis to identify the degree to which each group of symptoms inform different levels of syndrome severity.

## Method

### Participants

The present study was conducted in an outpatient research center at the Instituto de Psiquiatria, Universidade Federal do Rio de Janeiro, Brazil, from November 2002 to November 2015. This was a naturalistic study and patients were not specifically recruited to take part in it, but were already under regular treatment there. Not all the patients were involved in the study at the same time, and they were not necessarily assessed during the entire 13-year period. The inclusion criteria were a diagnosis of BD types 1 or 2, ≥ 18 years of age, written informed consent, and the occurrence of at least one manic or hypomanic episode, at least one depressive episode, and at least one period of euthymia during the course of the study. Notwithstanding, our study specifically addressed manic episodes. Depressive episodes and comparisons between the different phases of BD would be the object of other studies.

### Ethical considerations

This study was carried out in accordance with the Declaration of Helsinki and was approved by the local ethics committee.

### Instruments

Diagnoses of BD and affective episodes were established according to DSM-IV-TR criteria^13^ using the Structured Clinical Interview for the DSM (SCID).^14^ When a manic or hypomanic episode was detected, the Brazilian version of the Young Mania Rating Scale (YMRS)^15 , 16^ was routinely administered. The scale was not applied during episodes of depression or periods of euthymia.

The 11-item YMRS is one of the rating scales most frequently utilized to assess manic symptoms. It is based on the patient’s subjective report of his/her clinical condition and on an objective evaluation carried out by the examiner regarding the previous 48 hours. Each item is graded according to five levels of severity: seven items (elevated mood, increased motor activity-energy, sexual interest, sleep, language-thought disorder, appearance, and insight) are scored from 0 to 4 and the remaining four (irritability, speech, content, and disruptive-aggressive behavior) are scored from 0 to 8.^16^ Both the original scale^17 , 18^ and the version adapted for Brazilian Portuguese^19^ have shown high levels of validity and reliability.

Demographic and retrospective clinical data were also obtained while interviewing the patients.

### Procedures

Several patients had more than one manic or hypomanic episode during the clinical follow-up period, but only one episode per patient was considered in our study. We chose each patient’s episode with the highest total score on the YMRS. In case of a tie, the oldest episode was chosen.

The YMRS items were divided into three groups according to type of symptoms: mood, energy/activity, and other. The mood symptom group consisted of item 1 (Elevated Mood) and item 5 (Irritability). The energy/activity symptom group consisted of item 2 (Increased Motor Activity/Energy), item 3 (Sexual Interest), item 4 (Sleep), and item 6 (Speech – Rate and Amount). The other symptom group consisted of item 7 (Language-Thought Disorder), considered heterogeneous in terms of the changes evaluated, item 9 (Disruptive-Aggressive Behavior), related to both mood and energy, and item 8 (Content), item 10 (Appearance), and item 11 (Insight), not related to either mood or energy. This organization followed clinical criteria based on definitions of symptoms and the authors’ personal experience. Other studies that have compared energy or activity symptoms with mood symptoms also influenced this arrangement.^20 - 22^ Considering that each group of symptoms was composed of a different number of items, we calculated the average of each of these items to enable comparisons.

### Statistical analyses

Data were initially preprocessed to identify errors and inconsistencies. No outliers were removed and data were complete, with no missing cases. To verify which item groups were the most informative, a unidimensional version of the Samejima graded response model^23 , 24^ was used as an Item Response Theory (IRT) trait estimate.

The graded response model is an extension of the two-parameter dichotomous model developed for polytomous items. In this model, a single discrimination (slope) and several threshold (location) parameters are computed under the assumption that responses are graded on an ordinal scale.^25^ To identify the model, Ramsay acceleration was used along with the Broyden-Fletcher-Goldfarb-Shanno algorithm.^26^ After identification of the IRT model, the TIF was computed. The TIF is a measure of the amount of information provided by the item responses on a test about latent traits, which is computed by the sum of the information function of all the items. This statistic enables estimation of the degree to which such item groups contribute to understanding symptoms across the latent trait spectrum. The TIF returns Fisher information for the latent trait (θ) after summing item information functions. Pragmatically, TIF values range from -4 to 4 and a higher TIF indicates a better estimate of the precision of the results. Akaike (AIC) and Bayesian (BIC) information criteria, both crude and adjusted, were computed to determine the relative quality of the statistical models. Chi-square tests were used to check the association between the types of symptoms during the manic phase by comparing the area under the curve of the TIF results. All the analyses were performed using R 4.1 software and the mirt package.^26^

## Results

### Sample

During the study period, 243 patients were evaluated, but only 106 had at least one episode of mania or hypomania, at least one depressive episode, and at least one period of euthymia. None of the patients refused to participate in the study. There was no record regarding the number of times there was a manic or hypomanic episode but it was not possible to apply the clinical scale. Thus, a total of 106 manic or hypomanic episodes, one for each patient, were evaluated with the YMRS. The patients were followed for an average of 5.5 years (standard deviation [SD] = 3.1 years). There were 74 women (69.8%). The mean age of the participants was 52.5 years (SD = 11.7 years).

A total of 102 patients were classified as type 1 BD, and four were classified as type 2 BD. The mean age at the first episode of BD was 24.2 years (SD = 9.8 years). The total disease duration was an average of 24.1 years (SD = 12.6 years). Fifty-seven patients (53.8%) had been hospitalized at least once. The mean number of hospitalizations was 2.5 (SD = 4.0). Thirty-four patients (32.1%) had attempted suicide at least once. The mean number of suicide attempts was 0.8 (SD = 1.5). The average total score on the YMRS was 20.79 (SD = 8.44). There were no records regarding psychiatric comorbidities or the total number of manic and depressive episodes.

### TIF

The IRT model converged after 49 expectation maximization algorithm interactions. The AIC was 859.2018 and the BIC was 901.8169. These indices demonstrate the model’s preliminary adequacy.

**Figure 1:**
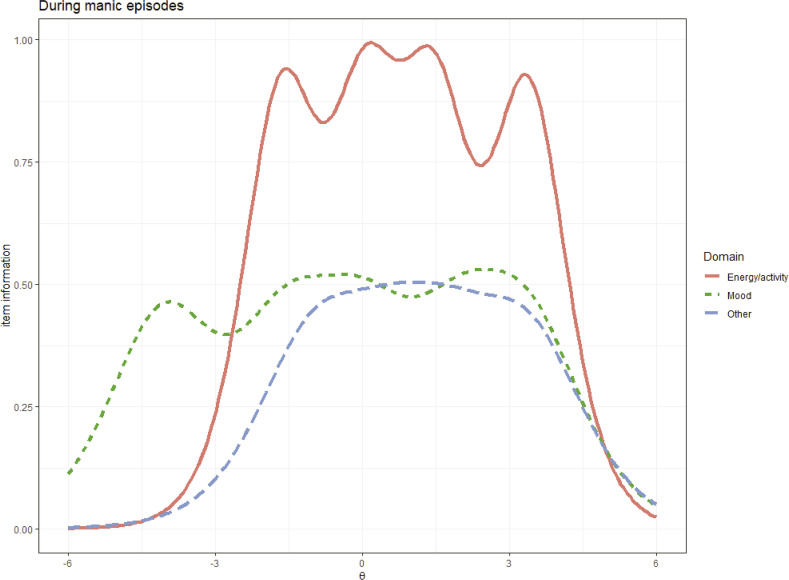
- Test information function (TIF) for the groups of symptoms during manic episodes

The discrimination parameters (i.e., slopes) were 1.34, 1.90, and 1.29, for the mood, energy/activity, and other groups respectively. The latent trait varied between -4 and 4. This index allows determination of how well the items discriminate different levels of the trait. Table 1 presents these results. TIF curves were computed for each of three suggested clinical classification criteria (mood, energy/activity, and other symptoms) during manic episodes ( Figure 1 ). Energy/activity symptoms were more essential during the manic phase of BD. The TIF curves with the highest peaks provided more information about one specific point in the latent trait, whereas the TIF curves with the longest lengths and peaks above the other curves were the most informative about the severity of one stage. The information regarding energy/activity represents 77% of the proportion of the total TIF and about 23% is related to the mood and other symptom groups. These two proportions are statistically different (χ^2^[1] = 30.42, p < 0.001), suggesting that the manic phase of BD is associated with symptoms related to energy/activity. The group of changes in energy or motor activity was only not the most informative at the extremes related to the severity of the manic episode, i.e., when the condition was very mild or very severe.

**Table 1:** Results of the IRT analysis using the GRM model with values for factor loadings, discrimination, and thresholds

Item groups	λ	a1	d1	d2	d3	d4	d5
Mood	0.619	1.34	5.415	2.014	-0.03	-2.693	-4.462
Energy/activity	0.745	1.903	3.12	-0.14	-2.76	-6.428	NA
Other	0.603	1.286	1.212	-0.692	-2.257	-4.29	NA
							
Full-information item factor analysis							
AIC = 859.2018; AICc = 865.3142							
BIC = 901.8169; SABIC = 851.2671							
SS Loadings: 1.302							
Proportion of variance: 0.434							

a1 = discrimination; AIC = Akaike information criteria; AICc = AIC with correction for small sample size; BIC = Bayesian information criteria; d = difficulty parameter; GRM = general regression model; IRT = Item Response Theory; NA = not available; SABIC = sample-size-adjusted BIC; SS = sum of squares; λ = standardized factor loadings.

## Discussion

Symptoms of manic episodes were assessed in 106 bipolar outpatients using the YMRS. The items on this clinical scale were divided into three groups according to clinical criteria: mood, energy/activity, and other. Using the TIF analysis, we found that energy/activity symptoms were the most informative in manic episodes. Compared to the mood changes and other symptom groups, the difference was statistically significant.

It should be noted that we observed that increased energy and motor activity were more informative than mood changes in manic episodes diagnosed according to DSM-IV-TR criteria.^13^ These criteria require presence of euphoria or irritability but not changes in the energy of motor activity. Thus, there was some sample bias in favor of mood changes in our study.

The average age of the patients was high and the average duration of the illness was relatively long. This may have occurred because of the inclusion criteria that required occurrence of at least one manic episode, one depression episode, and one period of euthymia. Furthermore, the study was carried out at a university hospital, where more severe patients are commonly referred to. It is not clear how these sample aspects might have influenced the results. Only two patients were diagnosed with type 2 BD. As the proportion between type 1 and type 2 patients was not known prior to the study, it was decided not to exclude any subjects from the sample.

A previous study^20^ also used a TIF analysis to establish comparisons between changes in mood and changes in energy/motor activity during an episode of bipolar mania and obtained results that were similar to the present one. However, unlike the present study, their sample comprised hospitalized patients and the assessment of manic symptoms was based on six items extracted from the Schedule for Affective Disorders and Schizophrenia-Changed version (SADS-C).^27 , 28^

Two other studies also investigated which symptoms are the most important in BD. Nevertheless, in contrast to the study with inpatients,^20^ these studies’ samples comprised outpatients, and, in addition to the manic phases, the depressive and euthymic phases were also evaluated. One of these studies^21^ used canonical discriminant analysis and found that items that evaluated increased motor activity (i.e., item 2 [increased motor activity/energy] on the YMRS and item 9 [agitation] on the Hamilton Depression Scale [HAM-D])^29^ were the items that best distinguished between mania, depression, and euthymia. In both scales, items that were related to mood symptoms were less important for this differentiation. The other study^22^ evaluated the same sample but applied only the HAM-D. According to their IRT analysis, items related to energy and motor activity were more informative than items related to mood.

Some factor analysis studies of manic symptoms have been performed. Bauer et al.^4^ evaluated a sample of patients with BD or unipolar depression and healthy controls. They found that “activation” was the factor most correlated with YMRS total scores. Akiskal et al.^3^ used the Beigel-Murphy Manic State Rating Scale (MSRS)^30^ in a sample of patients who were hospitalized with mania and obtained the same results. Benazzi and Akiskal^5^ applied the Mood Disorder Questionnaire (MDQ)^31^ in patients who were diagnosed with type 2 BD or unipolar depression and identified two main factors: “energized activity” and “irritability-racing thoughts.” Benazzi^32^ evaluated a similar sample and identified three factors: “elevated mood,” “mental activation,” and “behavioral activation.” In summary, in all four of these studies, factors that were related to increased energy/activity were among the most important, but only two of these studies identified a mood-related factor (irritability in one study and euphoria in the other).

Clinical practice also seems to reveal an association between hyperactivity and mania or hypomania. Benazzi^6^ found that hyperactivity was the most common symptom among patients with BD type 2, surpassing elevated mood, and was the symptom most associated with the diagnosis. Some authors^33 , 34^ have proposed that the presence of increased motor activity during a depressive episode would indicate occurrence of a mixed state. In a study with 7,689 patients who were currently experiencing a major depressive episode, Barbuti et al.^35^ found that psychomotor agitation had a stronger association with bipolar spectrum features than psychomotor retardation. Psychomotor retardation has classically been considered the cardinal symptom of depression.^36 , 37^

Motor activity can be objectively assessed by actigraphy. Some actigraphic studies show that patients with mania or hypomania have higher levels of motor activity than other depressed or euthymic bipolar patients, schizophrenia patients, and healthy controls.^7 - 10 , 38^ In contrast, other actigraphic studies have found a decrease in motor activity in patients with depression when compared with normal controls.^38 - 40^ They also found that remission of this change was associated with clinical improvement in the depressive symptoms.^41 - 43^

Other studies found that occurrence of elation or irritability did not clearly define a manic episode. In a review of 16 clinical studies of mania, Goodwin and Jamison^44^ found that a weighted average of 46% of manic patients presented sadness. In another review, Cassidy^45^ noted that anxiety was an especially common symptom in mixed mania episodes. According to Henry et al.^46 , 47^ mania is better characterized by the intensity of affective expression, which they referred to as hyperreactivity, than by mood quality (i.e., euphoria, irritability, or sadness).

Modification of the criteria for diagnosing a manic or hypomanic episode in the DSM-5^11^ significantly affected the clinical prevalence of BD. When the new criteria were applied to patients had previously been diagnosed with BD based on DSM-IV-TR criteria,^13^ the number of cases dropped by at least one third.^48 - 51^ Additionally, Fredskild et al.^50^ reported that patients who were diagnosed with a manic episode according to the DSM-5 criteria^11^ had higher scores on the YMRS compared with patients who met only the DSM-IV-TR criteria.^13^

Akiskal et al.^3^ proposed a more radical change to the diagnostic criteria of a manic episode, in which criterion A should include only psychomotor activation. Mood changes would be assigned to criterion B, but would not be restricted to elation and irritability (i.e., sadness and anxiety could also be accepted). The concept that changes in energy and motor activity would be more relevant than changes in mood could be extrapolated from BD, with at least three consequences from a nosographic perspective. The first consequence would be an even more radical change in the diagnostic criteria for mania, which aligns with the proposal presented by Akiskal et al.^3^ The second consequence would be a change in the criteria for the diagnosis of a major depressive episode, favoring motor changes over mood changes. Finally, BD and major depressive disorder would no longer be considered mood disorders and would instead be reclassified as energy and motor activity disorders. Obviously, these ideas are merely speculative and require additional scientific proof.

The present study has limitations. First, the choice of items that comprise each of the symptom groups (energy/activity vs. mood), while based on common observations in clinical practice, was arbitrary to some extent. The division of symptoms was not based on groups derived from factor analysis, which can certainly be criticized. Second, the choice of the YMRS may also be subject to criticism. The YMRS is the most widely used assessment of manic episodes, but it has significant psychometric problems. According to an IRT evaluation, several YMRS items provide little or no information.^52^ Another limitation was the decision to evaluate just the most severe episode of mania, since it may not be the most representative episode.

## Conclusion

The present results indicate that, on average, energy or motor activity changes tend to be more informative than mood changes in mania. Previous studies that made this comparison between these two symptom groups reported similar results.^21 , 22^ Results of factor analyses of manic symptoms, assessments of motor activity using clinical observation scales, and actigraphic studies point in the same direction.

## References

[1] Kraepelin E (1921). Manic-depressive insanity and paranoia.

[2] APA (1980). DSM-III: Diagnostic and Statistical Manual of Mental Disorders.

[3] Akiskal HS, Hantouche EG, Bourgeois ML, Azorin JM, Sechter D, Allilaire JF (2001). Toward a refined phenomenology of mania: combining clinician-assessment and self-report in the French EPIMAN study. J Affect Disord.

[4] Bauer MS, Crits-Christoph P, Ball WA, Dewees E, McAllister T, Alahi P (1991). Independent assessment of manic and depressive symptoms by self-rating. Scale characteristics and implications for the study of mania. Arch Gen Psychiatry.

[5] Benazzi F, Akiskal HS (2003). The dual factor structure of self-rated MDQ hypomania: energized-activity versus irritable-thought racing. J Affect Disord.

[6] Benazzi F (2007). Is Overactivity the core feature of hypomania in bipolar II disorder?. Psychopathology.

[7] Minassian A, Henry BL, Geyer MA, Paulus MP, Young JW, Perry W (2010). The quantitative assessment of motor activity in mania and schizophrenia. J Affect Disord.

[8] Perry W, McIlwain M, Kloezeman K, Henry BL, Minassian A (2016). Diagnosis and characterization of mania: Quantifying increased energy and activity in the human behavioral pattern monitor. Psychiatry Res.

[9] Perry W, Minassian A, Henry B, Kincaid M, Young JW, Geyer MA (2010). Quantifying over-activity in bipolar and schizophrenia patients in a human open field paradigm. Psychiatry Res.

[10] Kang GE, Mickey BJ, McInnis MG, Krembs BS, Gross MM (2018). Motor behavior characteristics in various phases of bipolar disorder revealed through biomechanical analysis: Quantitative measures of activity and energy variables during gait and sit-to-walk. Psychiatry Res.

[11] American Psychiatric Association (2013). Diagnostic and Statistical Manual of Mental Disorders, 5th edn (DSM-5).

[12] Petri E, Bacci O, Barbuti M, Pacchiarotti I, Azorin J-M, Angst J (2017). Obesity in patients with major depression is related to bipolarity and mixed features: evidence from the BRIDGE-II-Mix study. Bipolar Disord.

[13] American Psychiatric Association (2000). Diagnostic and Statistical Manual of Mental Disorder.

[14] Del-Ben CM, Vilela JAA, Crippa JA de S, Hallak JEC, Labate CM, Zuardi AW (2001). Confiabilidade da "Entrevista Clínica Estruturada para o DSM-IV - Versão Clínica" traduzida para o português. Rev Bras Psiquiatr.

[15] Vilela JAA, Crippa JAS, Del-Ben CM, Loureiro SR (2005). Reliability and validity of a Portuguese version of the Young Mania Rating Scale. Brazilian J Med Biol Res.

[16] Young RC, Biggs JT, Ziegler VE, Meyer DA (1978). A rating scale for mania: reliability, validity and sensitivity. Br J Psychiatry.

[17] Young RC, Nysewander RW, Schreiber MT (1982). Brief communication: Mania ratings at discharge from hospital: A follow-up. J Nerv Ment Dis.

[18] Young RC, Nysewander RW, Schreiber MT (1983). Mania scale scores, signs, and symptoms in forty inpatients. J Clin Psychiatry.

[19] Vilela JA, Crippa JA, Del Ben CM, Loureiro SR (2005). Reliability and validity of a Portuguese version of the Young Mania Rating Scale. Braz J Med Biol Res.

[20] Cheniaux E, Filgueiras A, Silva RDA Da, Silveira LAS, Nunes ALS, Landeira-Fernandez J (2014). Increased energy/activity, not mood changes, is the core feature of mania. J Affect Disord.

[21] Cheniaux E, Silva R de A da, Santana CM, Filgueiras A (2018). Changes in energy and motor activity: core symptoms of bipolar mania and depression?. Rev Bras Psiquiatr.

[22] Cheniaux E, da Silva R de A, Santana CMT, Nardi AE, Filgueiras A (2019). Mood versus energy/activity symptoms in bipolar disorder: Which cluster of hamilton depression rating scale better distinguishes between mania, depression, and euthymia?. Trends Psychiatry Psychother.

[23] Samejima F (1969). Estimation of latent ability using a response pattern of graded scores. Psychometrika.

[24] Samejima F, van der Linden WJ, Hambleton RK (1997). Handbook of Modern Item Response Theory.

[25] Krabbe PFM (2017). The Measurement of Health and Health Status.

[26] Chalmers RP (2012). mirt: A Multidimensional Item Response Theory Package for the R Environment. J Stat Softw.

[27] Spitzer RL, Endicott J (1978). Schedule of Affective Disorders and Schizophrenia-Change Version.

[28] Furlanetto LM, Bueno JB (1999). Depressäo em pacientes internados em hospital geral: validade do SADS em uma amostra brasileira. J Bras Psiquiatr.

[29] Hamilton MA (1960). A rating scale for depression. J Neurol Neurosurg Psychiatry.

[30] Beigel A, Murphy DL, Bunney WE (1971). The manic-state rating scale: scale construction, reliability and validity. Arch Gen Psychiatry.

[31] Hirschfeld RM, Williams JB, Spitzer RL, Calabrese JR, Flynn L, Keck PE (2000). Development and validation of a screening instrument for bipolar spectrum disorder: the Mood Disorder Questionnaire. Am J Psychiatry.

[32] Benazzi F (2004). Factor structure of recalled DSM-IV hypomanic symptoms of bipolar II disorder. Compr Psychiatry.

[33] Barroilhet SA, Ghaemi SN (2020). Psychopathology of Mixed States. Psychiatr Clin North Am.

[34] Pacchiarotti I, Kotzalidis GD, Murru A, Mazzarini L, Rapinesi C, Valentí M (2020). Mixed Features in Depression. Psychiatr Clin North Am.

[35] Barbuti M, Mainardi C, Pacchiarotti I, Verdolini N, Maccariello G, Angst J (2019). The role of different patterns of psychomotor symptoms in major depressive episode: Pooled analysis of the BRIDGE and BRIDGE-II-MIX cohorts. Bipolar Disord.

[36] Akiskal HS, McKinney WT (1975). Overview of recent research in depression. Integration of ten conceptual models into a comprehensive clinical frame. Arch Gen Psychiatry.

[37] Dantchev N, Widlocher DJ (1998). The measurement of retardation in depression. J Clin Psychiatry.

[38] Krane-Gartiser K, Henriksen TEG, Morken G, Vaaler A, Fasmer OB (2014). Actigraphic Assessment of Motor Activity in Acutely Admitted Inpatients with Bipolar Disorder. PLoS One.

[39] Burton C, McKinstry B, Szentagotai TA, Serrano-Blanco A, Pagliari C, Wolters M (2013). Activity monitoring in patients with depression: a systematic review. J Affect Disord.

[40] Cantisani A, Stegmayer K, Bracht T, Federspiel A, Wiest R, Horn H (2016). Distinct resting-state perfusion patterns underlie psychomotor retardation in unipolar vs. bipolar depression. Acta Psychiatr Scand.

[41] Raoux N, Benoit O, Dantchev N, Denise P, Franc B, Allilaire JF (1994). Circadian pattern of motor activity in major depressed patients undergoing antidepressant therapy: relationship between actigraphic measures and clinical course. Psychiatry Res.

[42] Volkers AC, Tulen JH, van den Broek WW, Bruijn JA, Passchier J, Pepplinkhuizen L (2002). 24-Hour motor activity after treatment with imipramine or fluvoxamine in major depressive disorder. Eur Neuropsychopharmacol.

[43] Todder D, Caliskan S, Baune BT (2009). Longitudinal changes of day-time and night-time gross motor activity in clinical responders and non-responders of major depression. World J Biol Psychiatry.

[44] Goodwin FK, Jamison KR (2007). Manic-Depressive Illness: Bipolar Disorders and Recurrent Depression.

[45] Cassidy F (2010). Anxiety as a symptom of mixed mania: implications for DSM-5. Bipolar Disord.

[46] Henry C, Swendsen J, Van den BD, Sorbara F, Demotes-Mainard J, Leboyer M (2003). Emotional hyper-reactivity as a fundamental mood characteristic of manic and mixed states. Eur Psychiatry.

[47] Henry C, M'bailara K, Desage A, Gard S, Misdrahi D, Vieta E (2007). Towards a reconceptualization of mixed states, based on an emotional-reactivity dimensional model. J Affect Disord.

[48] Grunze A, Born C, Fredskild MU, Grunze H (2021). How Does Adding the DSM-5 Criterion Increased Energy/Activity for Mania Change the Bipolar Landscape?. Front Psychiatry.

[49] Fredskild MU, Mintz J, Frye MA, McElroy SL, Nolen WA, Kupka R (2019). Adding Increased Energy or Activity to Criterion (A) of the DSM-5 Definition of Hypomania and Mania: Effect on the Diagnoses of 907 Patients From the Bipolar Collaborative Network. J Clin Psychiatry.

[50] Fredskild MU, Stanislaus S, Coello K, Melbye SA, Kjærstad HL, Sletved KSO (2021). Impact of modification to DSM-5 criterion A for hypomania/mania in newly diagnosed bipolar patients: findings from the prospective BIO study. Int J Bipolar Disord.

[51] Machado-Vieira R, Luckenbaugh DA, Ballard ED, Henter ID, Tohen M, Suppes T (2017). Increased activity or energy as a primary criterion for the diagnosis of bipolar mania in DSM-5: Findings from the STEP-BD study. Am J Psychiatry.

[52] Prisciandaro JJ, Tolliver BK (2016). An item response theory evaluation of the young mania rating scale and the montgomery-asberg depression rating scale in the systematic treatment enhancement program for bipolar disorder (STEP-BD). J Affect Disord.

